# Stem cell fate determination through protein O-GlcNAcylation

**DOI:** 10.1074/jbc.REV120.014915

**Published:** 2020-12-01

**Authors:** Muhammad Abid Sheikh, Bright Starling Emerald, Suraiya Anjum Ansari

**Affiliations:** 1 Department of Biochemistry, College of Medicine and Health Sciences, United Arab Emirates University, Al Ain, Abu Dhabi, UAE; 2 Department of Anatomy, College of Medicine and Health Sciences, United Arab Emirates University, Al Ain, Abu Dhabi, UAE; 3 Zayed Center for Health Sciences, United Arab Emirates University, Al Ain, Abu Dhabi, UAE

**Keywords:** O-GlcNAcylation, epigenetics, cell fate determination, gene expression, transcription, DON, 6-diazo-5-oxo-norleucine, ESCs, embryonic stem cells, HBP, hexosamine biosynthesis pathway, hPSCs, human pluripotent stem cells, HSCs, hematopoietic stem cells, iPSCs, induced pluripotent stem cells, MAP2, microtubule-associated protein 2, MHC, myosin heavy chain, MSCs, mesenchymal stem cells, NDDs, neurodevelopmental disorders, NTDs, neural tube defects, ODD, oxygen-dependent degradation, OGA, O-GlcNAcase, OGT, O-GlcNAc transferase, PPP, pentose phosphate pathway, PTM, posttranslational modifications, ROS, reactive oxygen species, SAM, S-adenosyl methionine, SOD, superoxide dismutase, TET, ten-eleven translocation

## Abstract

Embryonic and adult stem cells possess the capability of self-renewal and lineage-specific differentiation. The intricate balance between self-renewal and differentiation is governed by developmental signals and cell-type-specific gene regulatory mechanisms. A perturbed intra/extracellular environment during lineage specification could affect stem cell fate decisions resulting in pathology. Growing evidence demonstrates that metabolic pathways govern epigenetic regulation of gene expression during stem cell fate commitment through the utilization of metabolic intermediates or end products of metabolic pathways as substrates for enzymatic histone/DNA modifications. UDP-GlcNAc is one such metabolite that acts as a substrate for enzymatic mono-glycosylation of various nuclear, cytosolic, and mitochondrial proteins on serine/threonine amino acid residues, a process termed protein O-GlcNAcylation. The levels of GlcNAc inside the cells depend on the nutrient availability, especially glucose. Thus, this metabolic sensor could modulate gene expression through O-GlcNAc modification of histones or other proteins in response to metabolic fluctuations. Herein, we review evidence demonstrating how stem cells couple metabolic inputs to gene regulatory pathways through O-GlcNAc-mediated epigenetic/transcriptional regulatory mechanisms to govern self-renewal and lineage-specific differentiation programs. This review will serve as a primer for researchers seeking to better understand how O-GlcNAc influences stemness and may catalyze the discovery of new stem-cell-based therapeutic approaches.

The nutritional requirement of the developing embryo changes dramatically post fertilization, from the utilization of pyruvate and pyruvate analogs for energy production in the zygote to increased glucose uptake and glycolysis at the blastocyst stage (1, 2, 3). Such changes are required not only to meet the unique bioenergetic and biosynthetic demands during this critical period of development but also to provide cellular signaling to guide cell proliferation and differentiation programs. Glucose is the major source of energy and the source for the biosynthesis of macromolecules for the developing embryo at the blastocyst stage (4). Glucose metabolism is reported to play a key role during this stage of embryogenesis. Glucose also serves as a signaling molecule to govern the process of cell proliferation and differentiation (1, 5). The pluripotent stem cells at the inner cell mass of the blastocyst express glucose transporters, Glut1 and Glut3 at high levels for efficient glucose uptake (6). Furthermore, the expression of several key enzymes of glycolysis pathway is highly upregulated in these cells leading to aerobic glycolysis (Warburg effect), which may result in shunting of glycolytic intermediates to other pathways such as the pentose phosphate pathway (PPP) and hexosamine biosynthesis pathway (HBP). Once these pluripotent stem cells progressively differentiate into multipotent stem cells during organogenesis, the metabolic status of the cells gradually changes from glycolysis to oxidative metabolism (7, 8). This change in metabolic status is suggestive of the role of glycolysis in promoting rapid cell division, whereas oxidative metabolism is preferred in differentiating and more mature cells (9). What governs these preferences in metabolic states and whether cellular metabolism could also regulate stem cell fate decisions are questions currently under intense investigation. The emerging evidence suggests that nutrient signaling could play a key role in determining stem cell fate through epigenetic and gene regulatory mechanisms. It has been shown that the metabolic status of embryonic stem cells (ESCs) affects their epigenetic landscape and this impacts their tissue-specific differentiation potential (10, 11, 12) suggesting that cellular metabolism may not just be a passive state to support energy and biosynthetic demands of a cell but may also actively participate in governing cell fate decisions, possibly through epigenetic gene regulatory mechanisms. Thus, by providing metabolites that act as cofactors for histone and DNA modification enzymes and substrates for posttranslational modifications (PTMs) of transcription factors/epigenetic regulators, cellular metabolism could play a critical role in the regulation of the epigenome (11, 13). For example, acetyl CoA is utilized for protein acetylation, S-adenosyl methionine (SAM) is used as substrate for protein/DNA methylation, and NAD+/FAD/α-ketoglutarate act as cofactors of histone/DNA demethylases. Protein O-linkage of a β-N-acetylglucosamine (O-GlcNAcylation) is another such PTM that has gained attention in recent times. O-GlcNAcylation is regulated by HBP and is involved in a wide range of cellular functions including gene expression regulation (14, 15, 16). HBP is at the crossroads of all major metabolic pathways inside the cells including synthesis of carbohydrate, amino acids, nucleotides, and fatty acids as it utilizes substrates from these pathways, which include fructose-6-P, acetyl-CoA, glutamine, and UTP. Upon stimulation, HBP culminates through several steps in the production of UDP-GlcNAc, which provides the GlcNAc moiety for protein glycosylation and thus acts as a mechanism of nutrient signaling, which impacts various cellular processes (17, 18). Recent studies have started to demonstrate specific roles of protein O-GlcNAcylation in stem cell function during embryogenesis and in postnatal development using both stem cell culture experiments (ESCs and tissue resident adult stem cells) and animal models (19, 20, 21).

ESCs are pluripotent cells of the inner cell mass of the blastocyst stage embryo. These cells differentiate into all three germ layers and eventually develop into the embryo proper post implantation *in utero*. The standardization of methods to isolate ESCs from blastocyst and establishment of cell culture conditions for their maintenance (22) and their subsequent cell-type-specific differentiation have led to an explosion of studies utilizing established human and mouse ESCs to understand normal embryogenesis and developmental pathologies at cellular and molecular levels. Many of the fully differentiated adult tissues also contain multipotent/unipotent stem cells, which remain quiescent (G0) and enter into the cell cycle (G1) for proliferation/differentiation upon receiving cell-type-specific signal/stimuli. Examples of such adult stem cells include, but are not limited to, neural stem cells (NSCs) that can generate neurons, astrocytes, and oligodendrocytes; hematopoietic stem cells (HSCs) that generate blood and immune cells; mesenchymal stem cells (MSCs) that can give rise to cartilage, bone and fat cells; intestinal stem cells; and myogenic satellite cells. Most of these tissue-specific stem cells can also be isolated and cultured *in vitro* and have the ability of lineage-specific differentiation after receiving differentiation inducing growth factors/cytokines in the culture. Thus, similar to ESCs, tissue-specific adult stem cells are also utilized in a plethora of studies to understand their function.

Changes in metabolism due to conditions of extreme nutrient exposure such as hyperglycemia, dyslipidemia, or other metabolic perturbations could affect stem cell specification through nutrient availability or metabolite production. Protein O-GlcNAcylation, being sensitive to nutrient availability/metabolite levels, could regulate stem cell function through epigenetic/gene regulatory mechanisms during development and adult stages.

Therefore, in this review we will focus exclusively on the dynamic protein O-GlcNAcylation on stem cell function and O-GlcNAc-mediated epigenetic/gene regulatory mechanisms, which govern these functions. We will discuss the results of recent studies conducted in ESCs as well as adult stem cells combined with data from animal models on the effects of global O-GlcNAc levels on stem cell fate, the role of O-GlcNAcylation of key transcription factors in stem cell pluripotency and differentiation, and the role of other O-GlcNAc-mediated gene regulatory mechanisms. These discussions will not only provide up-to-date information on protein O-GlcNAcylation in stem cells and development but may also guide future studies with a focus on protein O-GlcNAcylation on pathological conditions, which deal with stem cell functions and metabolic perturbations such as the effects of diabetic pregnancy on the fetus.

## O-GlcNAcylation as a dynamic posttranslational protein modification

Protein glycosylation is one of the most diverse and complex co- and post-translational modifications of proteins. While the majority of glycans are added to the protein in the ER or Golgi complex, protein O-GlcNAcylation is cycled on and off the proteins in the nucleus, cytoplasm, and mitochondria (23, 24, 25, 26). Unlike other forms of glycosylation, O-GlcNAcylation is highly dynamic, and these marks are either added or removed to maintain homeostasis in response to diverse intra/extracellular signals (27, 28). O-GlcNAc transferase (OGT) is responsible for the addition of this mark whereas O-GlcNAcase (OGA) removes it from proteins (17, 29). Discovered initially by Hart and Torres in the early 1980s (30), protein O-GlcNAcylation has garnered much attention since then due to its role in various biological processes (15, 24, 31) and its association with pathologies such as cancer, diabetes, and neurodegeneration (32, 33, 34, 35, 36). Hundreds of proteins including histones and transcription factors are modified through O-GlcNAcylation in different cell types and biological contexts (26, 37). Histone modifications through O-GlcNAc in turn could regulate other epigenetic mechanisms such as DNA methylation, histone acetylation, and methylation, which are central to cell fate determination (16, 38, 39). Therefore, O-GlcNAcylation controls various aspects of cellular physiology including cell cycle progression, nutrient and growth factor sensing, response to different stresses, protein stability as well as localization (25, 40, 41) possibly through different mechanisms including the regulation of gene expression.

**Figure 1:**
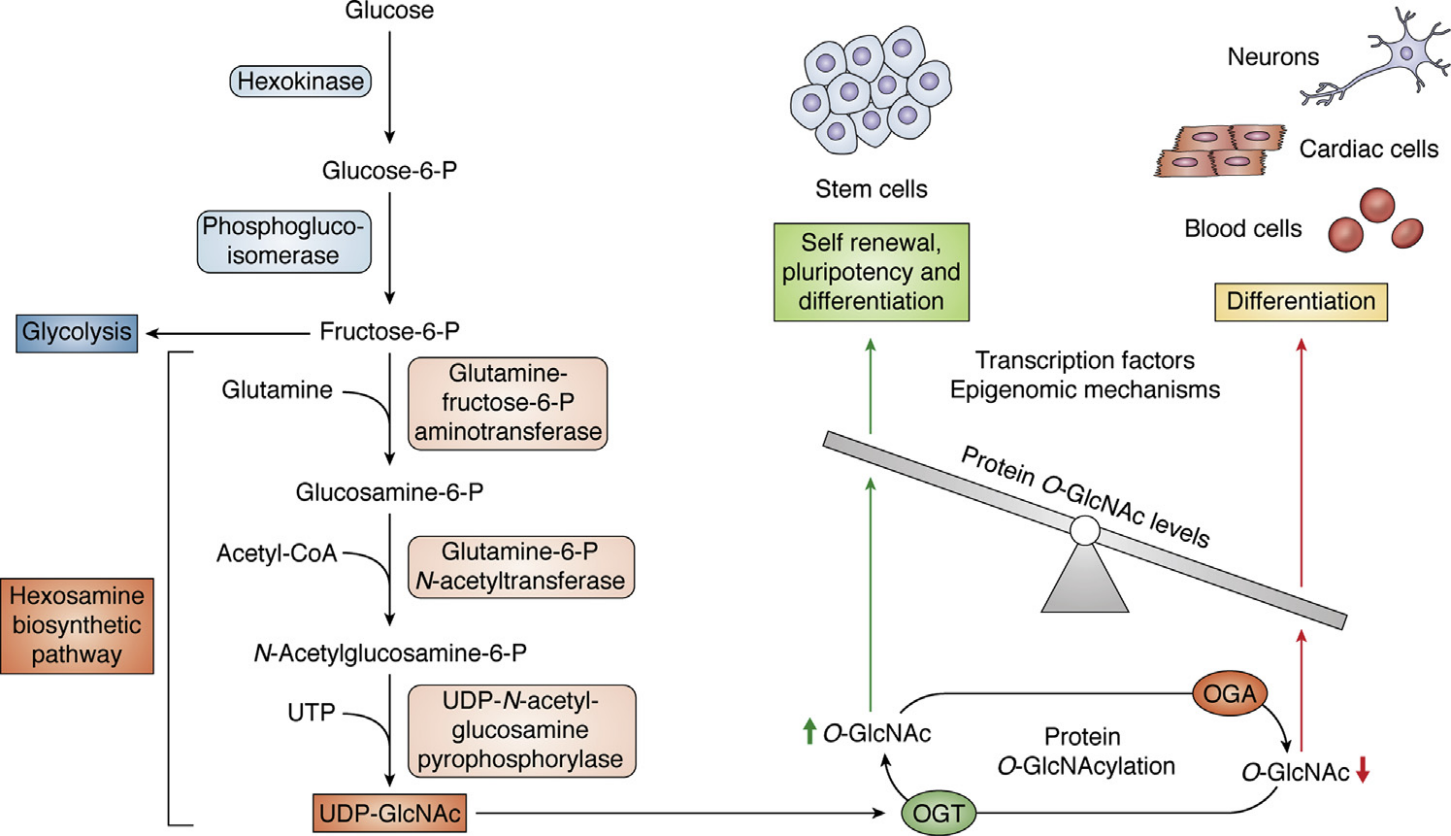
**O-GlcNAcylation regulates self-renewal, pluripotency, and differentiation in stem cells.** Some of glucose present inside the cells is directly channeled into HBP, where it is converted into UDP-GlcNAc, which is a key substrate for posttranslational modification of proteins through O-GlcNAcylation. The level of protein O-GlcNAcylation is determined through the dynamic activities of OGA and OGT enzymes, which in turn regulate self-renewal, pluripotency, and differentiation of stem cells *via* transcriptional and epigenomic control.

The influx through HBP depends upon the availability of glucose, glutamine, and acetyl-CoA in the cytoplasm of the cell (17, 42, 43, 44). Based on cell culture experiments using rat adipocytes, it is estimated that a small percentage (∼2–3%) of total cellular glucose entering inside the cell is metabolized by the HBP (44). However, this percentage could vary in a tissue/organ-specific manner as a recent study using *ex vivo* mouse heart perfusions has shown a much lower percentage (∼0.006% to ∼0.023%) of total glucose shunted into the HBP (45). The rate-limiting enzyme of the HBP pathway, glucosamine-fructose-6-phosphate aminotransferase isomerizing 1 (GFPT1), combines fructose-6-P from glycolysis and glutamine to form glucosamine 6-P, which is ultimately converted to UDP-GlcNAc in a series of steps and serves as a substrate for O-GlcNAcylation by OGT (46) (Fig. 1). Previous studies on various cell types and biological contexts have shown that changes in the availability of all three major nutrients, glucose, amino acids, and fatty acids, could modulate the levels of intracellular UDP-GlcNAc and HBP flux (47, 48, 49, 50, 51).

This suggests that O-GlcNAcylation could regulate protein function depending upon the nutritional environment of the cell, which depends upon the availability of glucose as it provides the first substrate for the synthesis of UDP-GlcNAc. However, the levels of UDP-GlcNAc and protein O-GlcANcylation may also depend on the levels of glutamine and acetyl CoA, which also feed into the HBP (52, 53). In support of this, cell culture experiments and studies in animal models have shown that an increase in glucose concentration leads to concomitant increase in O-GlcNAc levels (54, 55). For example, increasing glucose concentration (25 mM) in culture media of mesangial cells resulted in increased levels of O-GlcNAc as opposed to cells grown in media with normal level of glucose (5.5 mM) (56).

Nutrient conditions/glucose concentrations in culture media affect embryonic stem cell maintenance and subsequent differentiation efficiency to different lineages (1). Glucose deficient media favors development of early stage (during *in vitro* fertilization) human and mouse embryos (57) while higher glucosamine levels during *in vitro* maturation inhibit embryo development (58). Similarly, ESCs cultured in high glucose (25 mM) show reduced proliferation and oxidative stress (59), whereas culture of ESCs in physiological glucose level (5.5 mM) reduces oxidative stress and improves proliferative capacity (60). However, a high rate of glycolysis leading to increased acetyl CoA synthesis is needed for ESCs self-renewal (61), whereas supplementation of acetate could overcome glycolysis inhibition and loss of self-renewal suggesting that ESCs require acetyl CoA for proliferation, while reduced acetyl CoA levels lead to ESCs’ differentiation (62). Moreover, ESCs also rely on glutamine for growth and proliferation just like most cultured cell lines (63) as less than 5% of hESCs survived for 24 h in glutamine-depleted culture media (64).

This raises the possibility that the levels of glucose as well as flux through acetyl CoA and glutamine can all affect embryonic development and stem cell fate. Interestingly, all of these metabolites converge into UDP-GlcNAc production through HBP. This may also suggest that the enzymes OGT and OGA could be involved in stem cell fate regulation, possibly through O-GlcNAc-mediated transcriptional control of stemness genes. In the following sections, we discuss in detail the roles of O-GlcNAcylation in stem cell self-renewal, pluripotency, and differentiation, which are also summarized in Table 1.

**Table 1:** An overview of the role of O-GlcNAcylation in stem cell self-renewal, pluripotency, and differentiation

Yamanaka factors/Differentiation models	Type of cells	Protein/Global O-GlcNAc levels	Intervention	Biological impact	Ref.
SOX2	mouse ESCs	O-GlcNAcylation at S248	SOX2^WT^ to O-GlcNAc-deficient mutant SOX2 (SOX2^S248A^)	Increased somatic cell reprogramming, increased expression of pluripotency genes, altered genomic occupancy	(66)
OCT4	mouse ESCs	O-GlcNAcylation at T228	Point mutation at T228 to A228	Defective self-renewal, decreased pluripotency, altered expression of key regulatory genes that control pluripotency	(72)
Cardiogenesis	mouse ESCs	Decreased	6-diazo-5-oxo- norleucine (DON)	Decreased cardiomyocyte differentiation and cardiac-specific gene expression	(83)
Skeletal myogenesis	Mouse C2C12 cells	Decreased	OGA inhibition using siRNA or thiamet-G	Negative impact on skeletal myogenesis along with decreased expression of myogenic and muscle-specific markers	(84)
Erythrocyte differentiation	Mouse G1E-ER4 cells	Decreased	OGA inhibition using thiamet-G	Differential expression of erythroid-specific GATA-1 target genes leading to impaired erythropoiesis	(86)
Keratinocyte differentiation	Primary Cultures of human Epidermal keratinocytes	Decreased	Ectopic expression of OGA and OGT	Altered O-GlcNAcylation of a key transcription factor Sp1	(88)
Osteoblast differentiation	Mouse MC3T3-E1 preosteoblasts	Increased	OGA inhibition using thiamet-G and OGT inhibition using ST060266	OGA inhibition leads to increased differentiation while OGT inhibition suppresses differentiation	(90)
Adipocyte differentiation	mouse 3T3-L1 preadipocytes	Increased	GFPT1 inhibition using 6-diazo-5-oxo-orleucine	Blocked differentiation of preadipocytes	(94)
Chondrogenic differentiation	Mouse ATDC5 cells	Increased	OGA inhibition using thiamet-G	OGA inhibition mimicked the effect of insulin on ATDC5 cells resulting in increased expression of differentiation markers	(100)
Cortical Neurogenesis	Human ESCs	Decreased/Increased	OGT/OGA inhibition using thiamet-G and PUGNAc	Defects in progenitor proliferation and premature neuronal differentiation	(104, 106)

PUGNAc, O-(2-acetamido-2-deoxy-D-glucopyranosylidene)-amino-*N*-phenylcarbamate.

## O-GlcNAcylation in stem cell self-renewal and pluripotency

The key transcription factors that are known to control stemness and pluripotency include SOX2, OCT4, MYC, and KLF4 and are also collectively known as Yamanaka factors (65). These proteins either maintain stemness or promote differentiation in response to nutritional as well as microenvironment cues and are also used to reprogram somatic cells to induced pluripotent stem cells (iPSCs). It is interesting to note all of these Yamanaka factors except KLF4 are found to be O-GlcNAcylated, which in turn determines their functional significance (46). Myers *et al.*, 2016 reported that the O-GlcNAcylation of SOX2 at serine 248 (S248) is dynamically controlled in mouse ESCs. Upon differentiation of ESCs, O-GlcNAc occupancy of SOX2 at S248 is erased leaving this site unmodified. Replacement of wild-type SOX2 (SOX^WT^) with an O-GlcNAc-deficient mutant SOX2, where serine 248 was substituted for alanine (SOX2^S248A^), increases somatic cell reprogramming in mouse fibroblasts. Moreover, ESCs with mutant SOX2^S248A^ show increased expression of pluripotency genes and exhibit a decreased OCT4 requirement in maintaining pluripotency of ESCs. This suggests that O-GlcNAcylation at S248 of SOX2 has inhibitory effect on stem cell pluripotency (Table 1). The mutant form of SOX2^S248A^ also exhibited altered genomic occupancy with increased ability to bind SOX2 sites in the genome that are normally not bound by wild-type SOX2, suggesting that O-GlcNAcylation at S248 excludes it from several potential SOX2 binding sites in ESCs or increases its turnover. Since S248 lies in the transactivation domain of SOX2, it was not surprising that mutant form of SOX2^S248A^ also showed changes in its association with transcriptional regulatory complexes including its interaction with PARP1 (66). In ESCs, the interaction of SOX2 with PARP1 inhibits SOX2/OCT4 complex association with enhancers. This serves as a mechanism to fine-tune SOX2 activity and balances the maintenance of ESC pluripotency and differentiation (67). Therefore, SOX2 S248 O-GlcNAcylation is a requisite for this fine-tuning mechanism as SOX2 interacts with OCT4 and PARP1 in separate complexes. Loss of S248 O-GlcNAcylation in ESCs thus disrupts its association with both OCT4 and PARP1 and leads to its genome association independent of OCT4, thereby leading to increased expression of genes associated with pluripotency and a decreased requirement for OCT4 as observed (66).

Earlier studies that provided evidence that O-GlcNAc may regulate Oct4 included studies on the developmental effects of O-GlcNAcylation in zebrafish (68). OGT overexpression in zebrafish mimicked the phenotype seen in embryos deficient for Oct4 homolog spg/pou2 (69, 70, 71). Jang *et al.*, 2012 identified O-GlcNAcylation at threonine 228 on Oct4 protein purified from mouse ES cells and demonstrated a positive correlation between its transcriptional activity and the level of O-GlcNAc present on the protein. Mutation of T228 on Oct4 resulted in decreased O-GlcNAc levels at this site, which subsequently led to impaired reprogramming efficiency of fibroblasts as well as defective self-renewal of ES cells (Table 1). This suggests that O-GlcNAcylation of Oct4 is required for maintaining pluripotency in ES cells. By using mRNA expression analysis and serial chromatin-immunoprecipitation coupled with sequencing (ChIP-seq), the authors showed that O-GlcNAcylated Oct4 directly regulated transcription of 29 genes, including key regulators of pluripotency such as Nanog, Klf5, Klf2, Tbx3, Tcl1, and Nr5a2 (72). Another study confirmed that human OCT4 is positively regulated by OGT and identified several novel sites O-GlcNAcylated on OCT4 (73). Together, these findings provided evidence that OCT4 O-GlcNAcylation functions as a pluripotency regulator, adjusting the transcription of pluripotency-related genes in response to external stimuli.

Myc is another pluripotency gene and a known oncogene found to be overexpressed in various types of cancers. Although the role of Myc O-GlcNAcylation in pluripotency is not understood yet, Myc was found to be modified by OGT at Threonine 58 leading to its increased transforming activity and tumorigenicity in cancer cells (74). In prostate cancer, O-GlcNAcylation of c-Myc results in its stabilization leading to recurrence and poor prognosis (75). The expression of Myc is low in a normal resting cell; however, its expression is markedly increased upon mitogenic stimulation to transcriptionally control genes involved in cell proliferation and differentiation by dimerization with Max (76). It is important to mention that both pluripotent stem cells and many types of tumor cells rely on aerobic glycolysis, which is activated due to increased glucose uptake as well as conditions such as hypoxia. Shunting of some of this glucose to HBP in both cell types could provide signals for increased cell proliferation through protein O-GlcNAcylation of pluripotency genes such as Oct4 and c-Myc, whereas O-GlcNAcylation of SOX2 at S248 is inhibitory to pluripotency suggesting that O-GlcNAcylation of pluripotency-related transcription factors is complex and in need of further investigation to fully understand its role in normal and cancer cell self-renewal.

## O-GlcNAcylation in different models of stem cell differentiation

Protein O-GlcNAcylation plays a significant role during differentiation of stem cells and embryonic development as has been reviewed previously by Love *et al.*, 2010 (38). Deletion of OGT in mouse ESCs leads to cell death and is lethal to developing embryos (77). Deletion of OGT in other experimental models also showed similar results, for example, in zebrafish embryos, OGT is required for cell survival, maturation of oocytes, and epiboly movements (the movement and spreading out of cells into sheets of tissue especially during gastrulation) (68). Abnormal O-GlcNAc cycling led to changes in gene transcription and signaling pathways including insulin signaling during *Caenorhabditis elegans* embryonic development, and deletion of OGT significantly lowered their life span, whereas OGA deletion did not affect life span in the wild-type animals in this study (78, 79). Studies using conditional mouse mutants of OGT using a Cre-*loxP* system in thymocytes, neurons, oocytes, and fibroblasts found that it causes T-cell apoptosis, neuronal tau hyperphosphorylation, and fibroblast growth arrest with altered expression of several transcription factors with key roles in cell growth (80).

As described in the section above, O-GlcNAcylation regulates transcription factors involved in self-renewal and pluripotency of stem cells. Pluripotency genes, OCT4 and SOX2, harbor an O-GlcNAcylation mark, which is quickly erased following differentiation of ESCs (72). Pharmacological inhibition of OGA with GlcNAcstatin-C resulted in global increase in O-GlcNAcylation, which negatively regulated mouse ESCs differentiation into neural lineage possibly due to delay in the loss of Oct4 expression (81). On the other hand, OGA inhibitors, O-(2-acetamido-2-deoxy-D-glucopyranosylidene)-amino-*N*-phenylcarbamate (PUGNAc) and thiamet-G, did not affect the pluripotency or the differentiation potential of human pluripotent stem cells (hPSCs), although the excess O-GlcNAcylation due to OGA inhibition did alter the expression of lineage-specific genes (82). These studies further confirmed that higher level of O-GlcNAcylation is needed for the maintenance of self-renewal and pluripotency, while its reduction may be important for stem cell differentiation (Fig. 1). However, there may be differences in the role of O-GlcNAcylation in the differentiation of stem cells in a lineage-specific manner as discussed below in several examples of lineage-specific stem cell differentiation studies.

### mESCs-cardiac differentiation

OGT expression and global O-GlcNAc levels were markedly reduced upon cardiac differentiation of mouse ESCs and decreasing O-GlcNAcylation with an inhibitor of GFPT1, 6-diazo-5-oxo-norleucine (DON) positively contributed to the differentiation process. Thus, a decrease in protein O-GlcNAcylation not only enhanced cardiomyocyte development but is also essential for the normal differentiation of mESCs into cardiomyocytes (83) (Table 1).

### Skeletal myogenesis

An increase in the level of O-GlcNAcylation negatively regulated skeletal myogenesis. Studies using mouse myoblast cell line, C2C12, have shown that global levels of O-GlcNAcylation start to decrease during early myogenesis before the formation of myotubes. Genetic or pharmacological inhibition of OGA by thiamet-G to elevate O-GlcNAc levels resulted in decreased expression of Myosin heavy chain (MHC) genes and myoblast fusion, suggesting that a decrease in O-GlcNAcylation is essential for myoblast differentiation (84). OGA inactivation also resulted in decreased expression of myogenic regulatory factors Myogenin and Mrf4, which are required for the terminal differentiation of myoblasts (84, 85) as well as decreased expression of muscle-specific genes such as MHC, Caveolin 3, Troponin t1, and Troponin t2 suggesting that a decrease in O-GlcNAcylation is essential for the terminal differentiation of myoblasts (84) (Table 1).

### Hematopoietic stem cells–erythrocyte differentiation

O-GlcNAc homeostasis also contributes to cell fate decisions during erythrocyte differentiation of hematopoietic stem cells. Total O-GlcNAc levels were markedly reduced during erythropoietic cell differentiation and resulted in increased interaction between OGT, OGA, and GATA-1 (86), a master regulatory transcription factor of erythropoiesis (87) (Table 1). A total of 1173 genes were differentially expressed including 47 erythroid-specific GATA-1 target genes as a result of increased O-GlcNAcylation due to thiamet-G treatment during erythropoiesis leading to impairment of erythropoiesis.

### Myeloid and keratinocyte differentiation

Pharmacological inhibition of OGA by thiamet-G led to impaired myeloid cell differentiation (84). Similarly, a temporal decrease in the total O-GlcNAcylation was observed during calcium-induced keratinocyte differentiation (88). Sp-1 is an important transcription factor needed for the differentiation of keratinocytes (89). Although total Sp-1 levels were not altered during differentiation, O-GlcNAcylated Sp-1 was decreased. Ectopic expression of OGA resulted in increased transcriptional activity of Sp-1 in terms of Involucrin and Loricrin promoter activities, which are key genes of keratinocyte differentiation (88) (Table 1).

In contrast to the above examples where lineage-specific differentiation of stem cells was accompanied by a global decrease in O-GlcNAc levels, other examples of lineage-specific stem cell differentiation manifest increased protein O-GlcNAc levels during differentiation.

### Osteoblast differentiation

A global increase in the level of O-GlcNAcylation was reported during early stages of osteoblast differentiation of MC3T3-E1 preosteoblasts (90, 91). While OGA inactivation promoted differentiation, inhibition of OGT decreased the differentiation potential of MC3T3-E1 cells. Chemical inhibition of OGA resulted in increased expression of osteoblast-specific genes such as *alp* (alkaline phosphatase), *ocn* (osteocalcin), and *bsp* (bone sialoprotein) during differentiation (90, 92) (Table 1). Runx2, which is a key transcription factor of osteoblast differentiation, is modified by OGT, and inhibition of OGA resulted in its increased transcriptional activity (90, 92, 93). In addition, Ets1, another potent transcription factor of osteogenic genes, is also modified by O-GlcNAc, which results in upregulation of osteoblast differentiation. In contrast to this, inhibition of either OGA or OGT did not affect osteoclast differentiation of RAW264 cells (90).

### Adipocyte differentiation

An increase in the level of O-GlcNAcylation during differentiation of mouse 3T3-L1 preadipocytes was reported. During adipocyte differentiation, OGT expression was increased and correlated well with the increased expression of C/EBPα, which is a critical regulator of adipocyte differentiation (94, 95, 96) (Table 1). Following differentiation, several of the adipocyte-specific proteins were heavily O-GlcNAcylated including Vimentin and Nucleoporins, Pyruvate carboxylase, Long-chain fatty acyl-CoA ligase, and Ewing sarcoma protein. Among these, Vimentin is especially interesting as O-GlcNAcylation resulted in the conformational change from an extended fibrillar state to a complex cage formation during adipocyte differentiation (94, 97, 98). Pharmacological inhibition of the HBP pathway by GFPT1 inhibitor DON resulted in decreased levels of O-GlcNAcylation leading to impaired adipocyte differentiation confirming a critical role of O-GlcNAcylation in adipogenesis (94, 99).

### Chondrocyte differentiation

Global increase in protein O-GlcNAcylation was also observed during insulin-induced chondrogenic differentiation of ATDC5 cells. Both the expressions of OGT and OGA were elevated following differentiation. Inhibition of OGA by thiamet-G mimicked the effect of insulin as demonstrated by the increased expression of differentiation markers as well as increased activity of matrix metalloproteinases, MMP-2 and MMP-9 (100, 101). The MMP family of proteases play a crucial role in remodeling and degradation of extracellular matrix, which is a hallmark feature of chondrogenic differentiation (102, 103) (Table 1). *In vivo* administration of thiamet-G led to an increase in endochondral plate height (areas of cartilage located near the ends of bones where chondrocyte differentiation takes place) in newborn mice suggesting that O-GlcNAc elevation due to thiamet-G treatment leads to increased chondrocyte differentiation (100).

### hESC-neuronal differentiation

A global decrease in the levels of O-GlcNAc is observed during differentiation of hESCs toward neural fate in cell culture experiments as well as in mammalian brain during development (104, 105). Pharmacological inhibition of OGT during hESCs differentiation to neurons resulted in decreased protein O-GlcNAcylation on a global scale. Concomitantly, OGT inhibitor treatment during differentiation resulted in the expression of neuronal-specific markers β-III tubulin (TUJ1) and microtubule-associated protein 2 (MAP2) at an earlier stage than without treatment. Expression profiles showed that the genes involved in neuronal differentiation and forebrain development were significantly upregulated in OGT inhibited cells (106). However, a previous study from our lab (104) (Table 1) showed that sustained O-GlcNAc levels by thiamet-G and O-(2-acetamido-2-deoxy-D-glucopyranosylidene)-amino-*N*-phenylcarbamate treatments during *in vitro* differentiation of hESCs into cortical neurons also led to premature neuronal differentiation as well as reduced progenitor proliferation. In addition, thiamet-G treatment resulted in reduced AKT phosphorylation and increased apoptosis. We also noticed that the expression of neural progenitor markers, PAX6, OTX2, and SOX2, was reduced as reported in a similar study using same inhibitors of OGA (82). The expression of several genes involved in cortical neuronal differentiation such as NEUROD1, EOMES, and TBR1 was significantly upregulated and expressed earlier than normal during the course of differentiation, which could have resulted in premature neuronal production observed (104). These results suggest that both OGA and OGT are critical to *in vitro* human embryonic neurogenesis, and a perturbed O-GlcNAcylation leads to precocious neuronal production.

Animal experiments using a brain-specific OGA knockout mouse model showed delayed brain differentiation and neurogenesis as well as abnormal proliferation accompanying a developmental delay (107). Moreover, mESCs derived from Oga KO animals and WT littermates showed increased expression of the priming/predifferentiation factors Sox1, Nestin, and Otx2 as well as established neuronal markers including Mag, Aldh1, and Gfap in Oga KO mESCs compared with WT (107). However, long-term neuronal differentiation of Oga KO mESCs with retinoic acid showed dramatically reduced expression of neuronal marker β3-tubulin suggesting a perturbed neurogenesis due to elevated O-GlcNac levels. Thus, both *in vitro* neuronal differentiation of hESCs and *in vivo* mouse model confirm a perturbed neurogenesis due to perturbed O-GlcNAc levels.

One caveat of interpreting data from cells and tissues in which O-GlcNAc levels have been modulated globally is the homeostatic response of the O-GlcNAc cycling enzymes. Of note, abundance of both OGT and OGA appears to be linked to global O-GlcNAc levels. For instance, use of OGA inhibitor thiamet-G led to increased expression of OGA and slightly reduced expression of OGT in several cell lines (108) and deletion of OGT in mouse embryonic fibroblasts led to a rapid decrease in OGA expression (109). Further exacerbating this concern are the proposed scaffolding roles of OGT and OGA, as well as the proteolytic role of OGT (110, 111, 112). Nonetheless, global manipulation of O-GlcNAc levels highlights a role for O-GlcNAc or the O-GlcNAc cycling enzymes, and studies of specific proteins, especially those with site-specific information, highlighted key roles for O-GlcNAc in impacting cell fate. However, it is important to assess the direct effect of glucose/nutrient perturbations to understand precisely how acute or chronic nutrient perturbations affect O-GlcNAc-dependent cell fate changes as it could still be postulated that excess glucose affects stem cell fate decisions during embryogenesis or in adult stem cells due to altered O-GlcNAc levels. In agreement with this, the role of nutrient perturbations such as maternal hyperglycemia on embryopathies is well established (113, 114). Associations between maternal hyperglycemia and neurodevelopmental disorders (NDDs) is also being increasingly recognized (115), and animal models of maternal hyperglycemia have shed light on the defective neurodevelopmental mechanisms (116). A direct role of O-GlcNAcylation on neural tube malformation in diabetic pregnancy has been reported recently (117). The authors reported elevated O-GlcNAc levels in the embryos of diabetic mice and in neural stem cells cultured in high glucose leading to increased reactive oxygen species (ROS) due to stimulation of mitochondrial superoxide production and programmed cell death in these cells. Moreover, inhibition of OGT with ST045849 significantly reduced ROS levels. They also noticed that neural tube defects (NTDs) in diabetic mice were much higher compared with control animals and treatment of diabetic mice with OGT inhibitor (ST045849) significantly reduced the NTD rate and a decrease in apoptosis. Thus O-GlcNAcylation may have a direct role in embryopathies associated with maternal hyperglycemia. The role of hyperglycemia-mediated O-GlcNAcylation on osteogenic differentiation of mouse C2C12 by BMP2 in cell culture has also been reported (118). High glucose or glucosamine led to decreased osteogenic differentiation due to reduced expression of Runx2 and other osteogenic genes. Moreover, high-glucose-suppressed osteogenic differentiation could be alleviated by OGT inhibitor, ST045849. These studies thus clearly establish a key role for protein O-GlcNAcylation in stem cell fate determination in both embryonic and tissue-specific adult stem cells as a result of nutrient perturbations. The next question is how could perturbed O-GlcNAcylation affect stem cell fate decisions apart from inducing ROS generation and apoptosis? It is well established that unique transcription factor network and epigenetic mechanisms control stem cell fate decisions of proliferation/differentiation during development and adult life (119, 120). Thus, it is possible that protein O-GlcNAcylation could control such transcriptional/epigenetic mechanisms and thus affect stem cell fate. Indeed, several studies have reported protein O-GlcNAcylation on many epigenetic regulators and histone proteins confirming its role in epigenetic gene expression regulation, which we discuss in detail in the next section.

## Role of O-GlcNAcylation on epigenetic regulation of stem cell fate

**Figure 2:**
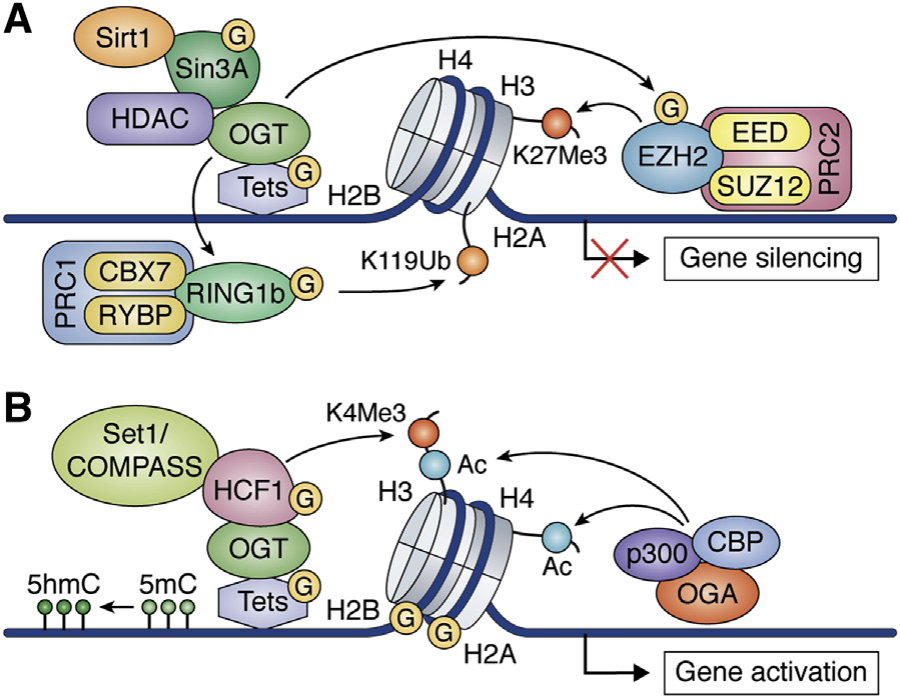
**O-GlcNAcylation cross talk with different epigenetic modifiers to regulate gene transcription.***A*, OGT is targeted to promoters by TETs to recruit transcriptional repressors Sin3A, Sirt1, and HDACs to repress gene transcription. In addition, O-GlcNAcylation of RING1B by OGT at sites that are present in a region that controls the binding of RING1B to the two components of PRC1 (RYBP and CBX7) results in H2BK119 ubiquitination leading to silencing of a specific subset of genes. O-GlcNAcylated form of RINGB1 is present preferentially near genes related to neuronal differentiation of ES cells. In the PRC2 complex, EZH2 is modified by O-GlcNAcylation at Ser75, which results in it being stabilized. All of this cross talk between O-GlcNAcylation and different proteins converges onto transcriptional repression. *B*, O-GlcNAcylation of TET proteins results in the conversion of 5 mc to 5 hmc. OGT also modifies HCF1, which in turn recruits Set1/COMPASS to carry out H3K4me3 resulting in gene activation. At certain gene promoters, OGA recruits p300 and CBP resulting in the acetylation of H3 and H4, which results in transcriptional activation.

Protein O-GlcNAc modifications are shown to regulate self-renewal, pluripotency, and differentiation of stem cells through epigenetic mechanisms. An increase in the level of O-GlcNAcylation leads to upregulation of genes that are silenced by epigenetic mechanisms in ESCs suggesting the involvement of O-GlcNAcylation in histone modifications and DNA methylation (81). OGT has been shown to modify epigenome marks by regulating Polycomb-group (Pc-G) proteins and Ten-Eleven Translocation (TET) enzymes, which in turn regulate transcription of genes involved in stem cell differentiation and embryonic development. TET proteins have enzymatic activity to carry out oxidation of 5-methylcytosine (5 mC) to 5-hydroxymethylcytosine (5 hmC) in DNA (121) (Fig. 2). 5hmc is predominantly present at sites where transcription is initiated, on gene promoters with bivalent chromatin, and within transcriptionally active genes. Active (H3K4me3) and repressive (H3K27me3) histone marks present in bivalent chromatin are crucial features of gene expression regulation during cell fate determination (39). The enzymatic activity and subcellular localization of all TET proteins (TET1, TET2, and TET3) are regulated by OGT as all TET proteins are found to be heavily O-GlcNAcylated (40). At least six O-GlcNAcylation sites have been reported for Tet1, while both Tet2 and Tet3 might have up to 20 sites for O-GlcNAcylation in competition with phosphorylation (122). TET proteins recruit OGT to chromatin and enhance its enzymatic activity as downregulation of TET proteins resulted in decreased O-GlcNAcylation of chromatin proteins (123, 124, 125). In ESCs, OGT influences histone methylation, including H3K4me3, and DNA methylation through TET1 enzyme, and recruitment of other transcription factors to specific target gene promoters (39, 126, 127, 128). TET1 is highly expressed in ESCs and is required to maintain pluripotency. TET1 was found to be O-GlcNAcylated by OGT in mESCs and depletion of OGT led to reduced TET1 and 5hmc levels on TET1 target genes, whereas overexpression of OGT had opposite effects suggesting that O-GlcNAcylation positively regulates TET1-dependent 5hmc levels and target gene expression (127). In addition to its enzymatic function, TET1 is responsible for recruitment of chromatin repressor complexes, polycomb repressor complex 2 (PRC2), and Sin3A to developmental genes in ESCs and thus helps to maintain pluripotency through transcriptional repression of these genes (129). *In vitro* studies had also shown direct interaction of OGT with Sin3A through its Tetratricopeptide repeat (TPR) domain (130). Thus, through either O-GlcNAcylation of TET1 or direct interaction with repressor Sin3A, OGT plays a critical role in transcriptional repression of developmental genes (127) and maintenance of pluripotency.

In another example, O-GlcNAcylation of Host cell factor 1 (HCF-1) by OGT led to its proteolytic cleavage and activation. In the process of proteolytic maturation of HCF-1, one of the six centrally located 20 to 26 amino acid sequence repeats called HCF-1_PRO_ is cleaved by OGT in the presence of UDP-GlcNAc (111). HCF-1 is known to control cell cycle by interacting with a number of transcription factors as well as epigenetic regulators including HDAC/Sin3A and histone H3 lysine 4 methyltranseferase (H3K4 HMT) complexes, which regulate transcriptional repression and activation respectively (131, 132) (Fig. 2). TET2 or TET3-OGT interaction, by promoting O-GlcNAcylation of HCF-1, helps to recruit SET1/COMPASS complex and H3K4Me3 and subsequent transcriptional activation in ESCs (123) (Fig. 2). In addition to ESCs, O-GlcNAcylation is also found to be involved in neuronal fate determination through the interaction of OGT with TET3, which results in the recruitment of NeuroD1 to brain-specific genes (133). Thus TET-OGT interactions might have much broader role in epigenetic gene regulation by their interaction and O-GlcNAcylation of HCF1, Sin3A, or other transcription factors in different complexes. These complexes may function in a cell type and context-dependent manner such as nutrient fluctuations or expression levels of OGT/OGA in different cell types. Moreover, O-GlcNAcylation also regulates gene expression through direct modification of polycomb repressive complex 1 and 2 (PRC1 and PRC2) proteins. These proteins are essential for repression of homeotic genes, which are master regulators of body plan during embryonic development (134). The catalytic subunit of PRC1 (RING1B/RNF2) carries out ubiquitination of H2A on Lys119 (H2AK119Ub) leading to transcriptional repression (135). In human ESCs, O-GlcNAcylation of RING1B was reported on two sites (Ser278 and Thr250/Ser251) (136), which are involved in the binding of RING1B to CBX7 and RYBP (PRC1 components involved in gene repression) (137, 138) (Fig. 2). O-GlcNAcylation of RING1B was decreased during neuronal differentiation of human ESCs (136). The O-GlcNAcylated form of RING1B is preferentially located near genes required for neuronal differentiation, whereas non-O-GlcNAcylated RING1B preferentially binds to metabolic and cell cycle genes. This means increased O-GlcNAcylation of the catalytic subunit of PRC1 represses genes required for neuronal differentiation of hESCs, whereas those motifs were absent on genes that control self-renewal and pluripotency including cell cycle genes (136). Similarly, Enhancer of zeste 2 (EZH2), which is the catalytic subunit of PRC2, carries out trimethylation of histone H3 on Lys27 (H3K27me3) resulting in chromatin silencing (139). OGT interacts with EZH2 causing O-GlcNAcylation at Ser75 leading to stabilization of EZH2 (Fig. 2). Both OGT and EZH2 are overexpressed in breast cancer cells, which negatively regulates tumor suppressor genes (140). However, OGT knockdown in mouse ESCs did not affect the levels and activity of EZH2 showing the link between OGT and EZH2 might be cell type dependent. In addition, mouse ESCs deficient in core subunits of PRC2 showed reduced levels of O-GlcNAcylation raising the possibility that PRC2 is involved in controlling normal levels of OGT in mouse ESCs (141).

O-GlcNAc-dependent epigenetic mechanisms also influence neuronal subtype specification during ESCs differentiation (142). During neuronal differentiation, an epigenetic change involving histone acetylation and DNA methylation at orexin gene leads to either orexin or nonorexin neurons and is regulated by O-GlcNAcylation. Orexin, also known as hypocretin, is a neuropeptide encoded by the HCRT gene and is involved in wakefulness, feeding, and other behaviors. OGT colocalizes with Sirt1, Sin3A, and Ezh2 to form a repressor complex at the Hcrt locus, which ultimately leads to the inactivation of Hcrt gene leading to the production of nonorexin neurons. On the other hand, OGA interacts with HAT p300 and CBP at the Hcrt gene leading to its activation, which ultimately results in the production of orexin neurons (142) (Fig. 2). These studies establish that OGT/OGA in association with epigenetic regulators regulate neuronal subtype specification during development. However, cell fate determination through O-GlcNAcylation could also depend on its roles during cell division due to direct O-GlcNAcylation of histone proteins. O-GlcNAcylation of histones has been reported on all four histones, H2A, H2B, H3, and H4, which could be in competition with phosphorylation for site occupancy (53, 143). For example, the O-GlcNAc/phosphorylation competition on histone H3 has been reported to be essential for the regulation of mitosis. OGT overexpression leads to reduction of phosphorylation at Ser-10 of H3 leading to impaired chromosomal segregation, while G2-M transition is impaired by OGA inhibition (144, 145). Furthermore, O-GlcNAcylation of H2B at Ser-112 is increased in response to double-stranded DNA breaks to provide genomic stability during the cell cycle (146).

In summary, the examples discussed above clearly demonstrate the involvement of OGT/OGA and protein O-GlcNAcylation in epigenetic regulation in different models of stem cells. Based on these studies, it can be speculated that OGT along with TETs and other corepressor proteins including Ezh2 and Sin3A or through direct O-GlcNAcylation of histones on promoters of developmental genes helps to keep them repressed, while other O-GlcNAc mechanisms simultaneously help promote cell proliferation such as OCT4 O-GlcNAcylation in ESCs. During differentiation, reduction in the levels of O-GlcNAc may lead to derepression of developmental genes causing cell differentiation. However, this explanation seems too simplistic as O-GlcNAc levels during cell differentiation are not always reduced, instead changing in a lineage-specific manner as discussed above. Thus, the effect of metabolic perturbations on cell fate is lineage and stage-specific and may utilize some of these epigenetic gene regulatory mechanisms resulting in pathological development. Thus, the effect of nutrient perturbations on O-GlcNAc-dependent epigenetic dysregulation during development needs to be further explored in a cell type/lineage-specific manner.

## O-GlcNAcylation of gene-specific transcription factors needed for cell differentiation

**Figure 3:**
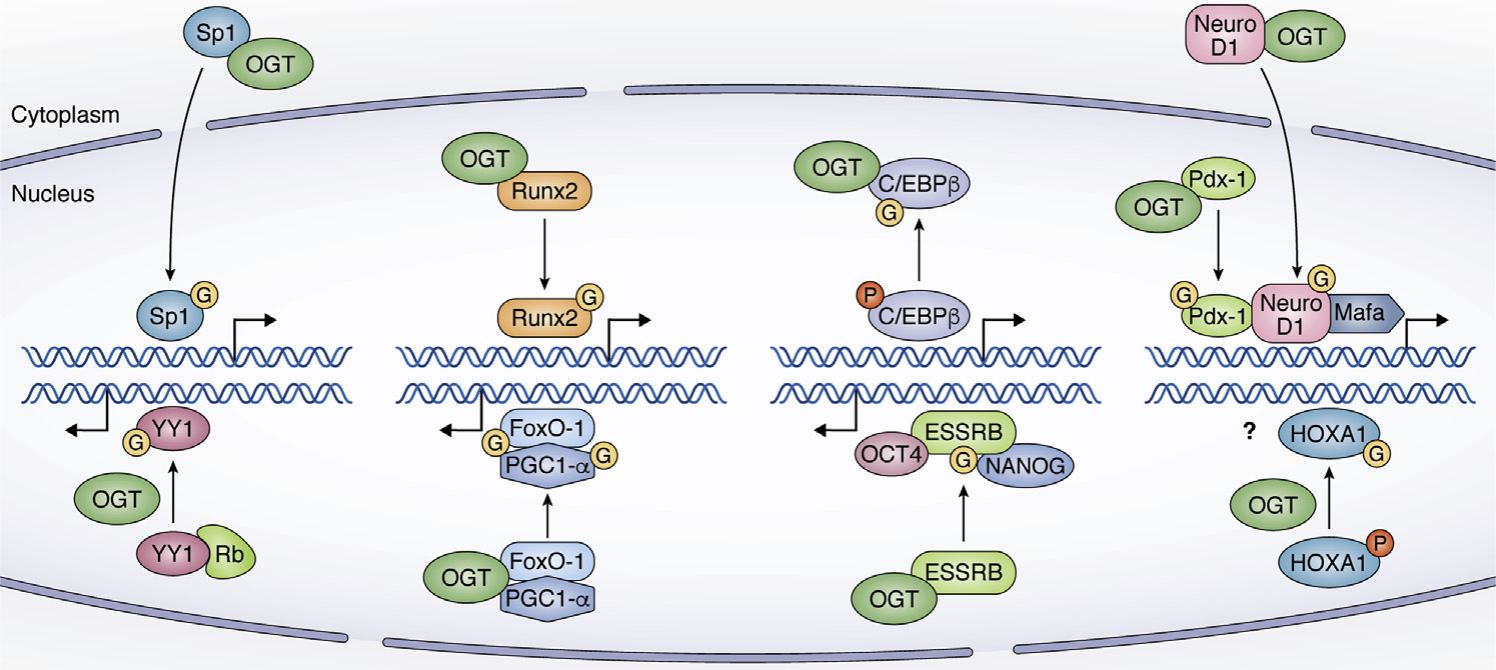
**O-GlcNAc modifications of transcription factors affect their activities in different ways.** Interaction of OGT with Sp1 results in its O-GlcNAcylation leading to nuclear localization and enhanced transcription. Runx2 is O-GlcNAcylated during osteogenic differentiation of MSCs, which results in increased activity of its transcriptional targets. The phosphorylated form of C/EBPβ is bound with DNA to carry out expression of adipogenesis-related genes. However, O-GlcNAcylation of C/EBPβ in competition with phosphorylation interferes with its function leading to reduced expression of its target genes. O-GlcNAcylation of NeuroD1 by OGT causes its translocation from the cytoplasm to the nucleus. The DNA-binding capability of Pdx-1 is enhanced following O-GlcNAcylation. High glucose is also linked to increased expression of MafA. Binding of retinoblastoma protein (Rb) with YY1 inhibits its recruitment to DNA. O-GlcNAcylation of YY1 inhibits its interaction with Rb, thus increasing the ability of YY1 to bind DNA and activate transcription. O-GlcNAc modification of coactivator PGC-1α results in the recruitment of OGT to FoxO1 promoting its O-GlcNAcylation and subsequent activation. ESSRB is modified by OGT resulting in its stabilization and enhanced transcriptional activity by interacting with OCT4 and NANOG to regulate pluripotency in mouse ESCs. HOXA1 exists in both phosphorylated and O-GlcNAcylated forms. However, the function of HOXA1 O-GlcNAcylation is not clear.

The proteins found to be O-GlcNAcylated include a large number of DNA-binding transcription factors (TFs), and the modification affects their transcription, localization, stability, DNA binding, and protein interaction activities (Fig. 3). Along with the O-GlcNAcylation of pluripotency associated TFs, OCT4, SOX2, and c-MYC as discussed in the sections above, several other gene-specific TFs are found to be O-GlcNAcylated and thus could regulate stem cell fate in addition to the regulation of pluripotency. The first O-GlcNAc-modification of a transcription factor was reported for Sp1, which is ubiquitously expressed (147). Sp1 is responsible for the regulation of those genes that contain GC-rich promoter sequences. Sp1 O-GlcNAcylation has been reported to control its stability, nuclear localization, and interaction with other regulators of transcription in a tissue-specific manner. For example, in liver cells, insulin induces Sp1 phosphorylation and O-GlcNAcylation leading to nuclear accumulation and subsequent increase in the expression of gene targets of Sp1 (148) (Fig. 3). However, Sp1 O-GlcNAcylation also causes transcriptional repression (149), which could occur through its interaction with OGT and repressor mSin3A (130) or due to inhibition of O-GlcNAcylated Sp1 association with transcription activators such as TAF110 (150), Oct1 (151), Elf1 (152), and NF-Y (153).

Similar mechanisms of O-GlcNAc-dependent gene regulation as observed for Sp1 were also noticed for TFs with a role in cell fate determination. For instance, HBP affects the activity of FOXO family of transcription factors, which are modified by O-GlcNAcylation (154, 155). FOXOs help in the maintenance of genomic and protein integrity by preventing accumulation of ROS in stem cells by inducing the transcription of ROS scavenger genes, superoxide dismutase (SOD), catalase, and others (156). An earlier study using rat hepatoma cell line showed that PGC-1α interacts with OGT to target FOXO1, which led to its enhanced O-GlcNAcylation and subsequently increased transcriptional activity (157) (Fig. 3). O-GlcNAcylation of FOXM1 regulated its expression in breast cancer, and a decrease in O-GlcNAc levels resulted in decreased cell cycle progression as well as increased expression of p27kip1, a cell cycle inhibitor gene (158).

HBP pathway also regulates hypoxia-inducible factor (HIF1a). Prolyl hydroxylases (PHDs) are α-ketoglutarate (αKG)-dependent dioxygenases, which hydroxylate HIF1α in its oxygen-dependent degradation (ODD) domain allowing its proteasomal degradation while αKG provides electrons in this process and is decarboxylated to succinate. Thus, the lack of molecular oxygen or unavailability of αKG leads to the inhibition of PHDs and the consequent stabilization of HIF1α (159). OGT influenced HIF1a stability by controlling levels of αKG in cells as decreasing O-GlcNAcylation in cancer cells increases αKG and HIF-1α hydroxylation, leading to enhanced proteasomal degradation (160). McGinn *et al.*, 2017 (161) showed that HIF1a also regulates self-renewal genes in pancreatic cancer, and its downregulation led to decreased stemness phenotype.

Likewise, the N-terminal domain of HOXA1 was found to interact with and to be modified by OGT. HOXA1 belongs to the HOX family of homeodomain transcription factors involved in the regulation of gene transcription during development extending from embryonic patterning to cellular differentiation (162). HOXA1 can be both phosphorylated and O-GlcNAcylated on its peptide sequence 146 AGGTVGSPQYIHHSY 160. The phosphorylation takes place at either Thr149 or Ser152, while O-GlcNAcylation always occurs at Thr149. However, O-GlcNAcylation of HOXA1 did not affect its stability, subcellular localization, and transcriptional activity (Fig. 3) as assessed by a plasmid-based reporter assay (162); thus whether HOXA1 O-GlcNAcylation affects its endogenous activity in terms of binding to chromatin DNA and transcriptional activation needs further investigation. Moreover, this study was performed in cancer and immortalized cell lines, thus the role of HOXA1 O-GlcNAcylation needs investigation in stem cells or in other developmental models.

Another example of a TF with a role in metabolism and cell fate is NeuroD1, a bHLH transcription factor expressed in neurons as well as in beta cells of pancreas where it is required for endocrine cell differentiation and pancreatic development (163). NeuroD1 binds to the E-box of the insulin gene promoter to stimulate its transcription by recruiting another transcription factor E47 (163). Increased glucose concentration results in an increase in insulin gene transcription by combined action of NeuroD1, Pdx-1, and MafA (164, 165) (Fig. 3). Glucose levels also control the localization of NeuroD1 within the cell (166). Under low or normal glucose concentration, NeuroD1 is primarily present in the cytoplasm. However, an increase in glucose concentration results in shuttling of NeuroD1 from cytoplasm to nucleus to activate transcription of insulin gene, which is dependent on the O-GlcNAc modification of NeuroD1. OGT inhibition using siRNA resulted in decreased nuclear localization of NeuroD1, whereas pharmacological inhibition of OGA led to nuclear localization of NeuroD1 in the absence of high glucose to regulate insulin gene transcription (166).

Yin Yang1 (YY1), which regulates transcription of a number of genes involved in cellular growth, differentiation, and replication, is another example of a transcription factor found to be O-GlcNAcylated. Deletion of YY1 gene results in embryonic lethality in mouse suggesting it is essential for development (167). Glucose exposure increases O-GlcNAc modification of nuclear YY1, which occurs independently of the cell type as it was observed in both actively proliferating and quiescent cells (168). During the G1 phase, YY1 binds to hypophosphorylated Rb protein, which leads to inhibition of YY1 binding to DNA. However, the interaction between Rb and YY1 is disrupted upon O-GlcNAcylation of YY1 leading to its association with target gene promoters (Fig. 3). Thus, the O-GlcNAcylation of YY1 is dependent on the levels of glucose inside the cells, and high glucose causes YY1 O-GlcNAcylation and its dissociation from Rb, whereas under low glucose conditions, non-O-GlcNAcylated YY1 is bound with Rb protein suggesting that O-GlcNAcylation could affect cell cycle by regulating binding of YY1 and Rb (168).

However, as mentioned, all of these studies were performed in either cancer cell lines or immortalized primary cells and whether such O-GlcNAc-dependent transcriptional regulation by these TFs occurs in normal stem cell proliferation and differentiation remains to be seen. On the other hand, there are examples where the role of O-GlcNAcylated TFs is investigated in stem cells. For instance, C/EBPβ, which belongs to bZIP transcription factors, is involved in adipocytic differentiation with increased expression during adipogenesis (169, 170). Phosphorylation of C/EBPβ at Thr-179, Ser-184, and Thr-188 is required for its activation and binding to DNA (171, 172). However, O-GlcNAc modification at Ser-180 and Ser-181 inhibits the essential neighboring phosphorylation leading to its inactivation and subsequently delays adipogenesis (95) (Fig. 3). Site-directed mutagenesis to replace Ser-180 and Ser-181 with alanine resulted in increased transcriptional activity of C/EBPβ *via* enhanced phosphorylation. This clearly showed that O-GlcNAcylation interferes with the function of C/EBPβ in competition with phosphorylation to regulate adipocyte differentiation (95).

Similarly, TF, Runx2, which is a master regulator of osteoblast differentiation, was found to be O-GlcNAcylated during osteogenic differentiation of bone-marrow-derived MSCs (Fig. 3) and OGA inhibition led to significantly enhanced osteoblast differentiation of MSCs as evidenced by the activity of alkaline phosphatase (ALP), an early marker of bone formation and a transcriptional target of Runx2 (92). ESSRB, a critical transcriptional regulator of pluripotency, was found be to O-GlcNAcylated by OGT on serine 25 resulting in its stabilization and increased transcriptional activity by facilitating its interaction with OCT4 and NANOG in mESCs (173) (Fig. 3). These studies clearly support the notion that nutrient sensing by gene and tissue-specific transcription factors through O-GlcNAcylation could be a key mechanism to regulate gene expression, cell proliferation/differentiation, and cell fate determination during both embryonic development and in adult stem cells. Further investigations to delineate the role of O GlcNAcylated TFs in stem cells need to be actively pursued.

## Conclusions and perspectives

**Figure 4:**
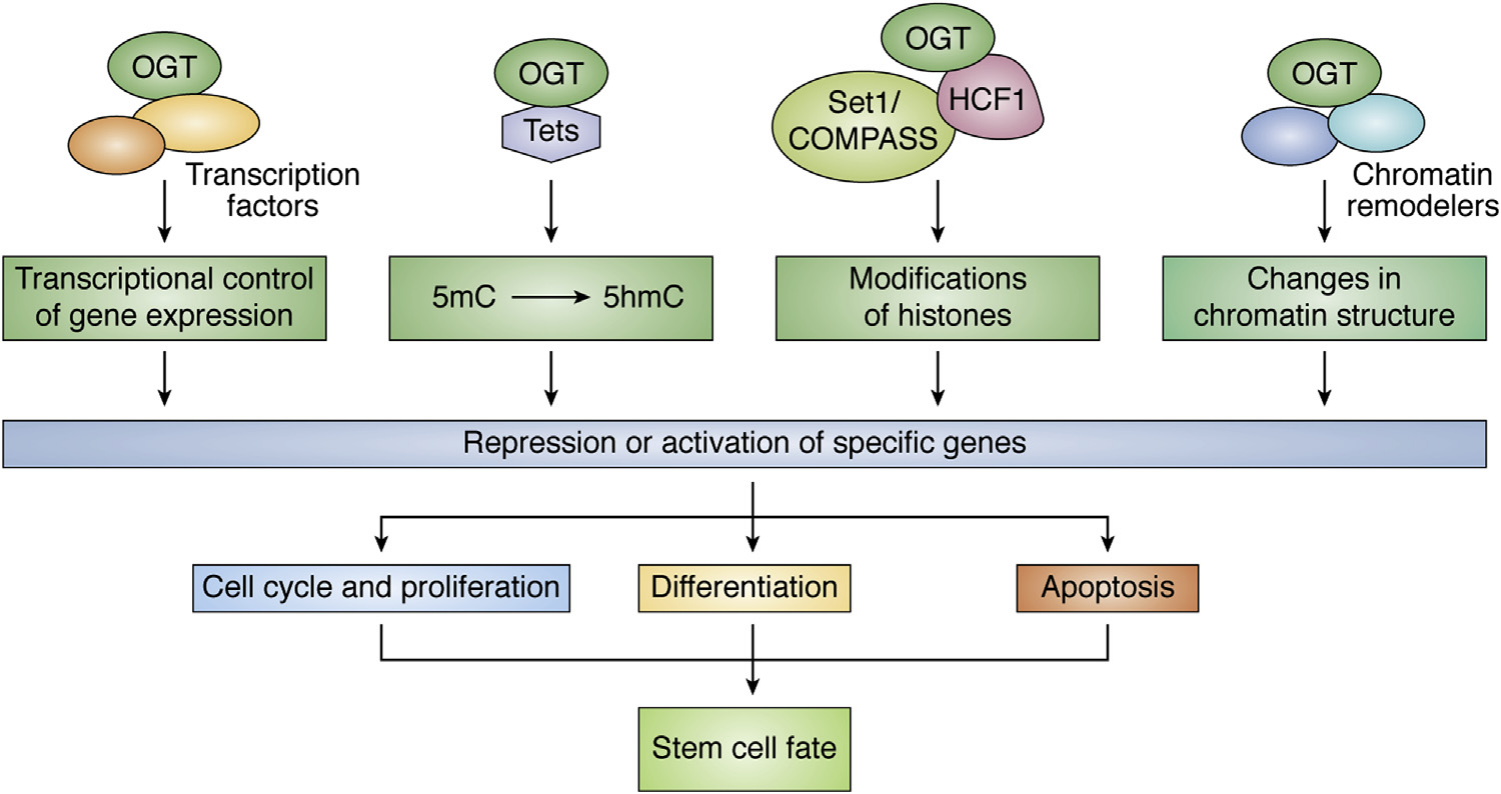
**Schematic diagram showing the interaction of OGT with different epigenetic modifiers and transcription factors to regulate transcription of genes that control stem cell fate through cell cycle and proliferation, differentiation, and apoptosis**.

O-GlcNAcylation of nucleocytoplasmic proteins is a dynamic PTM that is involved in the regulation of diverse cellular processes including stem cell fate. O-GlcNAcylation of proteins regulates self-renewal, pluripotency, and differentiation of stem cells through modifications of epigenetic regulators and transcription factors (Fig. 4). Deletion of OGT leads to embryonic lethality (77), whereas OGA deletion also leads to severe growth retardation of the developing embryo (174) suggesting that perturbations in the levels of O-GlcNAc are detrimental to embryogenesis. Nutrient fluctuations during embryogenesis such as maternal hyperglycemia could also perturb the levels of O-GlcNAc inside the cells of the developing embryo. Hyperglycemia in mothers during pregnancy due to pre-existing or gestational diabetes is associated with various types of embryopathies including NTDs, congenital heart defects, neurodevelopmental disorders, and macrosomia, among others (175). As hyperglycemia would lead to an increase in global O-GlcNAc levels, increased protein O-GlcNAcylation could thus be one major mechanism behind teratogenicity of glucose. The levels of glucose uptake and utilization are proportionately much higher in pluripotent stem cells and other highly proliferating cells. However, as the cells begin to differentiate, the uptake and utilization of glucose are reduced, which also results in reduced global O-GlcNAc levels during differentiation. Sustained O-GlcNAc levels during differentiation of pluripotent stem cells of the embryo could thus significantly affect cell fate specification involving various mechanisms of protein O-GlcNAcylation as we have discussed here. For instance, sustained O-GlcNAc levels during hESCs’ differentiation lead to reduced proliferation of neural stem cells (104) but increase the hPSC differentiation toward adipose-related mesoderm (82) suggesting differences in the mechanisms through which O-GlcNAc exerts its effect on lineage specification. This could possibly be due to cell-type-specific epigenetic mechanisms of O-GlcNAc-mediated gene regulation, which needs to be further explored.

Thus, future studies using animal models of maternal hyperglycemia or pluripotent stem cells with pharmacological/genetic manipulations of OGA/OGT enzymes are needed to identify lineage-specific epigenetic/gene regulatory mechanisms of sustained O-GlcNAc levels. This knowledge will be crucial in understanding not only the effects of maternal metabolic perturbations on embryonic development and associated pathologies but also vast majority of degenerative disorders related to metabolic syndrome or pathology of tumorigenesis, which is also associated with increased glucose uptake and utilization.

## Conflict of interest

The authors declare that they have no conflicts of interest with the contents of this article.
